# Decomposition and nutrient release from leaves of some common agroforestry tree/shrub species of Sudano-Sahelian West Africa

**DOI:** 10.1038/s41598-025-29117-9

**Published:** 2026-01-24

**Authors:** Siriki Fané, Deogratias Kofi Agbotui, Mariko Ingold, Cheikh Ndiaye, Sidi Sanogo, Andreas Buerkert

**Affiliations:** 1 Organic Plant Production and Agroecosystems Research in the Tropics and Subtropics (OPATS), University of Kassel, Steinstrasse 19, 37213 Witzenhausen, Germany; 2 Institut Polytechnique Rural de Formation et de Recherche Appliquée (IPR/IFRA) de Katibougou, Koulikoro, Mali; 3 College of Resources and Environmental Science, China Agricultural University (CAU), Beijing, China; 4 Centre Régional de Recherche Agronomique (CRRA) de Sikasso, Institut d’Economie Rurale (IER), Bamako, Mali

**Keywords:** Green manure, Nutrient cycling, Soil fertility, Litter quality, Shade trees, Tree species, Season, Plant ecology, Ecology

## Abstract

Foliar inputs from indigenous agroforestry tree/shrub provide organic matter and nutrients to sustain crop production in the West African Sahelian region. This study aims at monitoring the decomposition and nutrients release of leafy biomass of selected agroforestry tree and shrub species. In Mali and Burkina Faso, fresh leaves were collected from leguminous trees (*Faidherbia albida* (Delile) A. Chev., *Pterocarpus lucens* Lepr.) and non-leguminous trees (*Vitellaria paradoxa* C.F. Gaertn., *Khaya senegalensis* (Desr.) A. Juss.) and in Senegal, fresh leaves were obtained from leguminous trees (*Faidherbia albida* (Delile) A. Chev., *Pterocarpus lucens* Lepr. and *Piliostigma reticulatum* (DC.) Hochst.) and the non-leguminous tree *Guiera senegalensis* J.F. Gmel. The leaf samples collected during the dry and rainy seasons were deployed for decomposition and nutrient release experiments using the litter bag technique. Average decomposition rate for this study was 0.23 *k* week^− 1^ whereby the rate of decomposition was 155% higher in the rainy season than in the dry season. Average decomposition rate of leguminous tree and shrub leaves was 1.4-folds greater than that of non-leguminous counterparts. To optimize the synchronization of nutrient release from green manure with crop nutrient demands, manure should be co-applied considering seasonal and species effects.

**Supplementary Information:**

The online version contains supplementary material available at 10.1038/s41598-025-29117-9.

## Introduction

Subsistence agriculture employs 80% of the population in the Sahelian Region of West Africa^1^ thereby serving as the main source of food production in the region. Due to inherently poor soil fertility^2^, extended drought periods^3,4^, and rampant population growth, the Sahel is recurrently plagued by food insecurity^1^. The poor soils are subjected to further nutrient losses through crop harvest whereby extracted nutrients are insufficiently replaced due to unavailability and high cost of inorganic fertilizers^5,6^. Farmers in this region traditionally use leafy biomass of various trees and shrubs for improving soil fertility and mulching to retain soil moisture and catch Harmattan dust^7–9^. Commonly used species include *Vitellaria paradoxa* C.F Gaetn., *Parkia biglobosa* (Jacq.) R.Br. ex G. Don^2,10^, *Faidherbia albida*(Del.) A. Chev^2,11^., *Piliostigma reticulatum* (DC.) Hochst. and *Guiera senegalensis*J.F. Gmel^12,13^. Hence, there is great interest in using green manure in the form of pruned tree and/or shrub leaves as a soil amendment and nutrient source in the locally prevailing, small-scale low external input agricultural systems^14,15^. Upon application, decomposition and nutrient release processes transfer nutrients stored within this leafy biomass into the soil for crop use. Hereby decomposition and nutrient release processes are regulated by climate^16^, soil properties^17^, and biomass quality such as chemical properties of leafy biomass^14,18^. Studies by^14,19,20^ highlight that decomposition of leafy biomass is significantly faster during the rainy season than during the dry season. High moisture levels during the former increase soil organism activity, leading to faster mineralisation of organic matter and concomitant release of nutrients^19–22^. Nitrogen (N) and phosphorus (P) concentrations of tree leaves in Ethiopia^23^ and Nigeria^19^ were reported to increase during the rainy season reflecting an improved nutrient availability by accelerated litter decomposition and mineralization. Some studies found higher K and Ca concentrations in tree leaves of the dry season than of the rainy season^24,25^, which may result from physiological adaptation to drought and/or dust depositions from the Sahara desert^25,26^. In addition, leaf nutrient composition is also influenced by plant species, whereby nitrogen fixing leguminous trees, such as *F. albida*, *P. reticulatum*, and *Gliricidia sepium* (Jacq) Kunth ex. Walp, generally have higher N concentrations than non-fixing species. This results in lower C: N ratios of leaves, which is more favourable for microbial degradation. Previous work conducted in Nigeria^27^ and Burkina Faso^11^ found decomposition to be faster for leguminous trees compared to non-leguminous ones. On the other hand, mineralization of leafy biomass with low N concentration but high lignin concentration may lead to N immobilization, whereby soil microbes utilize nutrients from the soil solution and deplete available soil nutrients stocks^28,29^. Repeatedly, secondary metabolites such as lignin and polyphenols were found to retard the decomposition rate of tree leaves^28,30–33^. Such polyphenols, which are found in many sub-Saharan tree/shrub species like *F. albida* and *V. paradoxa*, have the ability to form complexes with N containing compounds, which subsequently slows down the N mineralization of polyphenol-rich leafy biomass^30^. Despite great interest in the use of tree leafy biomass as a nutrient source, decomposition and nutrient release studies of commonly used tree leafy biomass from the Sahelian region are few. Understanding the rate of leafy biomass decomposition and nutrient release is key for developing small scale low external input agricultural systems, where nutrient release is synchronized to crop demand for an efficient nutrient utilization. Hence this study aims to: (i) assess the seasonal variation in the chemical properties of leafy biomass from different tree and shrub leaves, (ii) investigate how seasonal variability influences the decomposition and nutrient release patterns of leafy biomass from different tree and shrub leaves, and (iii) examine the variation in decomposition and nutrient release patterns among different tree and shrub types. It hypothesises that: (i) the initial chemical properties of different tree/shrub leaves are different in the dry and rainy season; (ii) decomposition and nutrient release is faster during the rainy season than the dry season, (iii) decomposition and nutrients release are greater for leguminous tree/shrub (*F. albida*,* Pterocarpus lucens* Lepr. Ex Guill. & Perr. and *P. reticulatum*) than for non-leguminous (*Khaya senegalensis* (Desr.), *Guiera senegalensis* JF Gmel. and *V. paradoxa*).

## Results

### Initial chemical properties of tree and shrub leaves

There was significant interaction (*p* < 0.05) between country and leaf type for N, P, C/N ratio, and total phenols (Supplementary Table S1). Nutrient concentrations of collected leaves were similar across countries, whereas N, P, and Mg concentrations of leguminous tree/shrub were 73%, 85% and 38%, respectively, higher (*p* < 0.05) than concentrations in non-leguminous counterparts. In contrast, phenol concentrations in non-leguminous tree/shrub were two-fold higher (*p* < 0.05) than those in leguminous tree/shrub. Seasonality influenced nutrient concentrations with the dry season leading to 23% and 13% lower (*p* < 0.05) K and Mg concentrations i, respectively, in leaves than the rainy season (Supplementary Table S2). Phenol concentrations on the other hand were 37% lower (*p* < 0.05) in the rainy season than in the dry season.

In Senegal, *P. lucens* had the highest measured nutrient concentrations compared with the other species, and also the highest concentration of condensed tannins (Table 1). *P. lucens* had higher 55% higher N (*p* < 0.01), 21% higher P (*p* = 0.03), 38% higher K (*p* = 0.04), and 106% higher Mg (*p* = 0.04) concentrations than the average of the other species, resulting in a 1.5-fold lower C/N ratio than the other species. But also, it also had 319% higher CT concentrations than the average of *F. albida*,* G. senegalensis. F. albida* and *P. reticulatum*. For these three species C/N ratios were relatively similar, whereas CT concentrations differed. Condensed tannin concentration in *F. albida* was 12.7-times higher than that in *P. reticulatum*. Total phenol concentration in *G. senegalensis* was 10%, 29% and 70% higher (*p* < 0.01) than in *F. albida*, *P. lucens*, and *P. reticulatum*, respectively. However, the concentration of condensed tannins in *P. lucens* initial leaves, was 36-times greater (*p* < 0.01) than the lowest concentration in *P. reticulatum*.

**Table 1:** Initial chemical properties of tree/shrub leaves subjected decomposition study in Dahra, Sahelian zone of Senegal (*n* = 3).

Species	*N*	*P*	K	Mg	C: *N*	Total Phenols	Condensed-Tannin
	mg g^− 1^						
*F. albida*	19.35 (1.05) b	0.97 (0.01) b	6.74 (0.70)	3.13 (0.22) b	24.13 (1.86) b	38.70 (0.66) b	19.74 (1.31) b
*G. senegalensis*	15.46 (0.08) c	1.17 (0.05) ab	7.41 (0.07)	2.80 (0.05) b	31.31 (0.12) a	42.41 (0.08) a	18.51 (1.36) b
*P. lucens*	27.16 (0.42) a	1.43 (0.14) a	10.20 (4.10)	5.84 (1.46) a	17.95 (0.24) c	32.81 (0.64) c	55.53 (2.34) a
*P. reticulatum*	17.72 (0.68) b	1.44 (0.14) a	8.06 (0.32)	2.57 (0.09) b	26.02 (0.41) b	24.96 (0.36) d	1.55 (0.83) c
F value	58.98	4.91	0.52	4.22	32.94	108.20	145.59
*p*-value	< 0.01	0.03	0.68	0.04	< 0.01	< 0.01	0.01

Means in the same columns with different lower-case letters show significant differences in tree/shrub leaves (*p* < 0.05). Numbers in brackets after means of tree/shrub leaves show ± one standard error of the mean.

For dry season in Mali, nutrient concentrations in tree and shrub species differed among each another. While N, P, and K concentrations and C/N ratios of *F albida*, *K senegalensis*, did not differ significantly across seasons, *P. lucens* did (Table 2). *F. albida* had the highest N and P concentrations, which were 82% and 34% respectively, higher (*p* < 0.01) than the average of the other tree and shrub species while its K (*p* < 0.01) and CT concentrations (*p* < 0.01) and the C/N ratio (*p* < 0.01) were 21%, 51% and 41%, respectively lower than the average of other tree/shrub species. During the rainy season, average N concentration of *K. senegalensis* and *V. paradoxa* was 56% lower (*p* < 0.01) than the average of *F. albida* and *P. lucens*. Phosphorus concentration of *P. lucens* in the rainy season was 2-times greater than its P concentration in the dry season. In the dry season, the Mg concentration in *P. lucens* was 1.8-folds lower (*p* < 0.01) than that of *V. paradoxa*. In the rainy season, the Mg concentration in *K. senegalensis* was 2-folds lower (*p* < 0.01) than the average of *P. lucens* and *V. paradoxa*. Total phenol concentration in *V. paradoxa* was 68% and 271% higher (*p* < 0.01) than the averages of the other tree/shrub species in the dry and rainy season, respectively. In both dry and rainy season, condensed tannins concentration was 2-times higher (*p* < 0.01) in *K. senegalensis* than the average of the other tree/shrub species.

**Table 2:** Initial chemical properties of tree/shrub leaves subjected to a decomposition study in Koulikoro, Sudano-Sahelian zone of Mali (*n* = 3).

Season	Species	*N*	*P*	K	Mg	C: *N*	Total phenols	Condensed-tannin	
		mg g^− 1^							
Dry		16.58 (1.57) A	1.32 (0.15) A	9.51 (0.78) A	3.68 (0.27) A	30.41 (1.94) A	72.18 (6.67) A	42.62 (5.09) A	
	*F. albida*	25.07 (3.50) b	1.64 (0.00) ab	7.91 (0.02) ab	3.52 (0.01) abc	19.81 (101) a	59.82 (0.20) c	23.80 (0.17) a	
	*K. senegalensis*	12.30 (0.25) a	1.51 (0.39) ab	12.22 (2.71) abc	3.44 (0.54) abc	36.37 (0.61) cd	80.42 (0.99) d	69.86 (0.14) f	
	*P. lucens*	15.12 (0.33) a	0.59 (0.01) a	9.16 (0.14) ab	2.80 (0.05) ab	31.24 (0.50) b	44.82 (0.52) b	40.12 (0.12) e	
	*V. paradoxa*	13.84 (0.08) a	1.56 (0.02) ab	8.73 (0.10) ab	4.97 (0.06) c	34.23 (0.10) bc	103.66 (1.41) e	36.69 (0.29) d	
Rainy		20.80 (2.57) B	1.39 (0.17) A	11.51 (1.24) B	4.02 (0.33) A	27.10 (3.22) B	47.69 (10.06) B	30.23 (0.03) B	
	*F. albida*	26.99 (5.60) b	1.87 (0.45) b	6.90 (1.74) a	4.25 (0.67) bc	17.31 (1.84) a	27.60 (0.48) a	15.04 (0.75) b	
	*K. senegalensis*	12.85 (0.23) a	1.03 (0.09) ab	17.20 (0.27) c	2.28 (0.03) a	36.56 (0.42) cd	30.13 (0.24) a	34.86 (0.35) d	
	*P. lucens*	30.72 (2.28) b	1.78 (0.16) b	12.81 (0.55) bc	4.77 (0.08) c	15.81 (0.71) a	27.57 (0.84) a	42.32 (0.87) e	
	*V. paradoxa*	12.66 (0.11) a	0.87 (0.08) ab	9.12 (0.33) ab	4.77 (0.08) c	38.70 (0.27) d	105.44 (1.09) e	28.70 (0.85) c	
Two-way ANOVA									
	Season	F = 17.46 *P* < 0.01	F = 0.17 *P* = 0.68	F = 5.92 *P* = 0.03	F = 1.20 *P* = 0.29	F = 30.30 *P* < 0.01	F = 1761.58 *P* < 0.01	F = 682.86 *P* < 0.01	
	Species	F = 45.30 *P* < 0.01	F = 2.89 *P* = 0.07	F = 14.79 *P* < 0.01	F = 11.26 *P* < 0.01	F = 229.90 *P* < 0.01	F = 2778.29 *P* < 0.01	F = 860.67 *P* < 0.01	
	Season × Species	F = 14.48 *P* < 0.01	F = 7.22 *P* < 0.01	F = 2.87 *P* = 0.07	F = 9.76 *P* = 0.07	F = 50.62 *P* < 0.01	F = 359.14 *P* < 0.01	F = 280.74 *P* < 0.01	

Means along the same columns with different lower-case letters show significant differences between tree/shrub leaves and upper-case letters show significant differences between seasons (*p* < 0.05). Numbers in brackets after tree/shrub leaves means show ± one standard error of the mean.

In Burkina Faso in both, the rainy and the dry season, N, and P concentrations in *P. lucens* leaves were higher (*p* < 0.01) than in those of *F. albida*, *K. senegalensis*, and *V. paradoxa* (Table 3). Contrarily, total phenol concentrations in *V. paradoxa* leaves during both, the dry and rainy seasons were 48% and 304%, respectively higher (*p* < 0.01) than the average of other tree/shrub species. Average P concentration for all other leaves in the dry and rainy season was 5.5-times lower (*p* < 0.01) than P concentration in *P. lucens* in the rainy season. Whereas C/N ratio in both dry and rainy season in *P. lucens* leaves were 1.7-fold and 1.8-fold, respectively lower (*p* < 0.01) than in the average of the other tree and shrub species, its CT concentrations were 76% and 155% higher (*p* < 0.01) than that of the average CT concentration of the other tree and shrub species in both, the dry and rainy season. Average Mg concentration of *F. albida* and *K. senegalensis* in the dry season was 2-fold lower than Mg concentration in *F. albida* in the rainy season.

**Table 3:** Initial chemical properties of different tree/shrub leaves subjected to a decomposition study in Saria, Sudano-Sahelian zone of Burkina Faso (*n* = 3).

Season	Species	*N*	*P*	K	Mg	C: *N*	Total phenols	Condensed-tannin	
		mg g^− 1^							
Dry		15.45 (1.42) A	0.71 (0.07) A	6.41 (0.69) A	2.76 (0.26) A	33.45 (2.86) A	88.19 (6.13) A	44.73 (4.26) A	
	*F. albida*	17.54 (0.21) bc	0.72 (0.03) a	8.02 (0.35) bcd	2.36 (0.04) a	26.16 (0.12) b	97.12 (1.13) f	30.76 (0.52) c	
	*K. senegalensis*	11.12 (0.11) a	0.57 (0.08) a	3.26 (0.30) a	2.33 (0.21) a	41.46 (0.38) cd	75.28 (0.93) e	48.67 (0.23) e	
	*P. lucens*	21.94 (1.67) cd	0.96 (0.16) a	8.40 (0.01) bcd	2.50 (0.27) ab	22.23 (1.66) ab	63.80 (0.63) d	66.07 (1.08) g	
	*V. paradoxa*	11.19 (0.02) a	0.59 (0.13) a	6.89 (1.45) abc	0.84 (0.27) bc	43.96 (0.01) d	116.56 (0.65) g	33.43 (0.05) c	
Rainy		17.37 (1.64) B	1.67 (0.50) B	9.27 (0.89) B	3.42 (0.24) B	29.70 (2.38) B	52.60 (12.13) B	32.29 (5.59) B	
	*F. albida*	17.18 (2.14) bc	0.72 (0.06) a	5.14 (0.50) ab	4.72 (0.03) c	26.81 (3.25) b	17.24 (0.12) a	10.52 (0.01) a	
	*K. senegalensis*	12.36 (0.18) a	0.91 (0.08) a	10.13 (0.31) cd	2.52 (0.08) ab	37.86 (0.52) cd	40.42 (0.81) c	38.29 (1.34) d	
	*P. lucens*	25.89 (0.32) d	4.18 (1.10) b	10.04 (2.16) cd	3.30 (0.05) abc	18.77 (0.23) a	31.99 (0.44) b	59.34 (1.34) f	
	*V. paradoxa*	14.05 (0.18) ab	0.87 (0.05) a	11.76 (0.33) d	3.30 (0.05) ab	35.36 (0.36) c	120.75 (1.33) h	21.02 (0.62) b	
Two-way ANOVA									
	Season	F = 7.78 *P* = 0.01	F = 11.54 *P* = 0.04	F = 14.78 *P* = 0.01	F = 10.28 *P* = 0.01	F = 16.15 *P* < 0.01	F = 3630.69 *P* < 0.01	F = 451.78 *P* < 0.01	
	Species	F = 65.46 *P* < 0.01	F = 10.63 *P* < 0.01	F = 4.98 *P* = 0.01	F = 6.59 *P* = 0.01	F = 107.07 *P* < 0.01	F = 3026.78 *P* < 0.01	F = 1028.83 *P* < 0.01	
	Season × Species	F = 1.87 *P* = 0.18	F = 7.13 *P* < 0.01	F = 9.73 *P* < 0.01	F = 9.91 *P* < 0.01	F = 4.12 *P* = 0.02	F = 850.26 *P* < 0.01	F = 23.78 *P* < 0.01	

Means along the same columns with different lower-case letters show significant differences between tree/shrub leaves and upper-case letters show significant differences between seasons.

### Decomposition and nutrient release rates

The decomposition of leafy biomass was fastest during the initial 4 weeks after placement on the soil, whereby the decline in DM was considerably slower in Senegal compared with Mali and Burkina Faso. In both Mali and Burkina Faso, the total leaf DM disappeared from the litterbags after 15 weeks in the rainy season and after 32 weeks in the dry season, respectively.

**Fig. 1:**
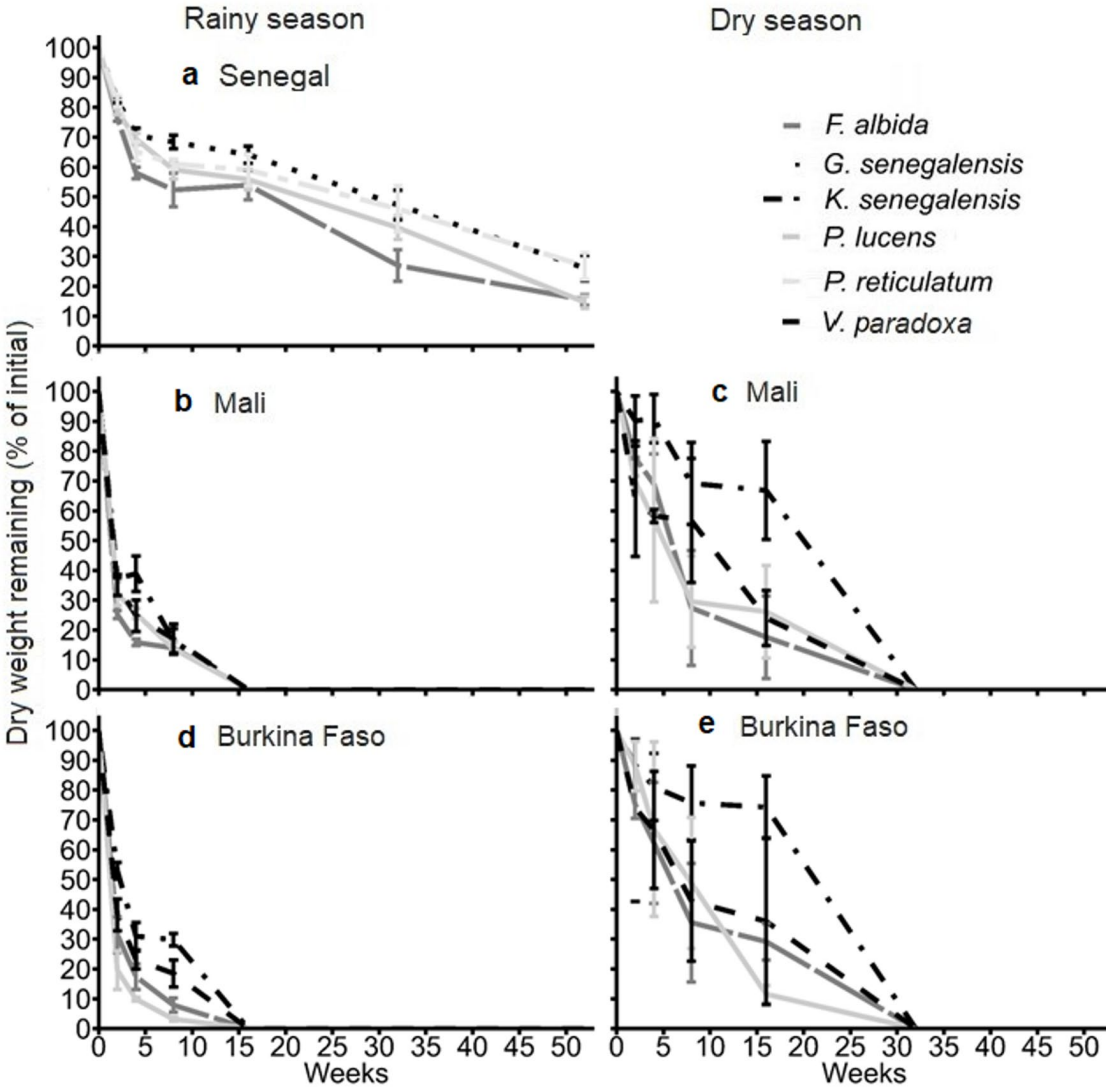
Decay patterns of different tree/shrub leaves after 52 weeks in litterbags placed in soil across season in Dahra in the Sahelian zone of Louga in Senegal, Katibougou and Saria in the Sudano-Sahelian zone of Koulikoro and West Central in Mali and Burkina Faso respectively. Error bars indicate +/− one standard error of the mean (*n* = 5).

In Senegal, decomposing leaves lost on average 30% of their initial dry weight in the first four weeks after placement (Fig. 1a and Supplementary Fig. S1). At weeks 2, 4, and 8 remaining DM of *G. senegalensis* were 26%, 30%, and 33%, respectively higher (*p* < 0.05) than those of *F. albida*. At 52 weeks after placement DM losses of *F. albida* and *P. lucens* were 46% and 51%, respectively, higher (*p* < 0.01) than that of *G. senegalensis*. In Mali, leaves lost 70% of their initial dry weight within the first four weeks after litterbag placement in the rainy season (Fig. 1b and Supplementary Fig. S2), which was two-folds greater than losses in the dry season (Fig. 1c). In the rainy season at weeks 2 and 4, *F. albida* DM losses were 19% and 38%, respectively, higher (*p* < 0.05) than losses of *K. senegalensis*. In the dry season, *P. lucens* DM losses at 4 weeks after placement was 376% higher (*p* < 0.05) than that of *K. senegalensis*. At 8 and 16 weeks, DM losses of *K. senegalensis* were 58% and 60%, respectively, lower (*p* < 0.05) than for *F. albida*, and 56% and 55%, respectively, lower than for *P. lucens*. In Burkina Faso, on average 30% of the initial dry weight of the decomposing leaves were lost during the first four weeks of the for dry season (Fig. 1d and Supplementary Fig. S3), which was 61% lower than in the rainy season (Fig. 1e). At 2 weeks after leaf placement DM losses of *F. albida* and *P. lucens* were 45% and 60% higher (*p* < 0.01) than those of *K. senegalensis* in the dry season. Similarly, at 4 and 8-weeks DM losses of *K. senegalensis* were 24% and 27%, respectively, lower (*p* < 0.01) than for *P. lucens* in the rainy season. At 16 weeks after leaf placement in dry season, *P. lucens* DM losses was 363% higher (*p* < 0.01) than that of *K. senegalensis*.

In Senegal, decomposition rate and release rates of N and P were significantly higher in leguminous species compared with non-legume species, whereas for K release rate constants were in a similar range except for *P. reticulatum* (Table 4 and Supplementary Table S6). The decomposition rate constant *k* of the legume species *F. albida* and *P. lucens* were by 100% higher than *k* of non-legume species *G. senegalensis* and *P. reticulatum* (*p* = 0.02). Similarly, the average N release constant of by *G. senegalensis* and *P. reticulatum* were 67% and 50% lower (*p* < 0.01) than of *F. albida* and *P. lucens*, respectively. Phosphorous release by *F. albida* and *P. lucens* were 200% and 100%, respectively higher (*p* = 0.04) than the average in *G. senegalensis* and *P. reticulatum*. Potassium release constant of *P. reticulatum* was 62%, 58%, and 55% lower (*p* = 0.03) than of *F. albida*, *P. lucens* and *G. senegalensis*, respectively.

**Table 4:** Mineralization rate constants (*k*: % day^− 1^) in the decomposition of leaves of leguminous tree/shrub species (*F. albida, P. lucens and P. reticulatum*) and non-leguminous *G. senegalensis* after 52 weeks in litterbags placed on the soil during the rainy season in Dahra in the Sahelian zone of Louga, Senegal (*n* = 5).

Species	Mass loss	*N*	*P*	K
*F. albida*	0.14 (0.04) a	0.21 (0.04) a	0.63 (0.22) a	0.91 (0.17) a
*G. senegalensis*	0.07 (0.01) b	0.07 (0.02) b	0.21 (0.01) b	0.77 (0.08) a
*P. lucens*	0.14 (0.01) a	0.14 (0.02) a	0.42 (0.09) ab	0.84 (0.15) a
*P. reticulatum*	0.07 (0.01) b	0.07 (0.01) b	0.21 (0.04) b	0.35 (0.07) b
F value	4.99	5.86	3.37	3.92
*p*-value	0.02	<0.01	0.04	0.03

Means in the same columns with different lower-case letters show significant differences between tree/shrub leaves (*p* < 0.05). Numbers in brackets after means of tree/shrub leaves show ± one standard error of the mean.

Overall, leguminous tree/shrub species (*F. albida* and *P. lucens*) had higher decomposition rate *k* and key nutrient release (N and P) than non-leguminous tree species (*K. senegalensis* and *V. paradoxa*), especially during the rainy season, in both Mali and Burkina Faso. Though non-leguminous showed slower decomposition and lower N and P release, they were similar in their K release, particularly during the rainy season.

In Mali, the trees/shrub leaves decomposition rates *k* were significantly influenced by season (*p* < 0.01) and species (*p* = 0.03). While N and P release rates were affected influenced by season (*p* < 0.01), species (*p* < 0.01), and their interaction (*p* < 0.05), K release rates were only affected by season (*p* < 0.01). Additionally, the decomposition rate *k* differed within species types, *P. lucens* had the highest decay constant in the dry season (0.25), while *F. albida* had the highest *k* in the rainy season (0.55). Average DM decomposition, and N, P and K release rates of tree/shrub leaves in the rainy season were 205%, 217%, 194%, and 225%, respectively higher (*p* < 0.01) than those of the dry season (Table 5 and Supplementary Table S7). During dry season *P. lucens* had 0.5–6.5-fold higher decay constants for DM loss, and N and P release compared with the other tree/shrub species. The decomposition rate of *F. albida* in the rainy season was 358% higher (*p* = 0.03) than that in the dry season. Similarly, the average decay constant of other tree/shrub during the dry season was four-folds lower (*p* < 0.01) than that of *F. albida*. Nitrogen and P release by *P. lucens* in dry season were 69% and 58%, respectively lower (*p* < 0.01) than those in the rainy season. While N and P release rates of *P. lucens* in the rainy season were 5.4 times and 5.8 times, respectively higher (*p* < 0.05) than the average of others tree/shrub leaves, its K release was 1.2-folds lower than the average of the other species in the dry season.

**Table 5:** Mineralization rate constants (*k*: % day^− 1^) in the decomposition of leaves of leguminous tree/shrub species (*F. albida* and *P. lucens*) and non-leguminous *K. senegalensis* and *V. paradoxa* after 52 weeks in litterbags placed across season in Katibougou in the Sudano-Sahelian zone of Koulikoro Mali (rainy, *n* = 5 and dry, *n* = 4).

Season	Species	Mass loss	*N*	*P*	K
Dry		0.96 (0.09) A	0.84 (0.32) A	1.17 (0.39) A	1.12 (0.32) A
	*F. albida*	0.84 (0.14) ab	0.84 (0.14) ab	0.91 (0.07) ab	0.98 (0.14) ab
	*K. senegalensis*	0.42 (0.07) a	0.42 (0.07) a	0.35 (0.00) a	0.84 (0.21) a
	*P. lucens*	1.75 (0.07) abc	1.19 (0.42) ab	2.10 (0.63) ab	1.75 (0.35) abc
	*V. paradoxa*	0.84 (0.07) ab	0.91 (0.63) ab	1.33 (0.84) ab	0.91 (0.56) a
Rainy		2.94 (0.40) B	0.40 (0.33) B	2.66 (0.40) B	0.33 (0.47) B
	*F. albida*	3.85 (0.35) d	3.36 (0.28) cd	4.62 (0.42) c	3.43 (0.42) cd
	*K. senegalensis*	2.10 (0.28) abc	1.33 (0.21) ab	1.61 (0.21) ab	3.85 (0.63) d
	*P. lucens*	2.80 (0.49) bcd	3.85 (0.42) d	4.97 (0.49) c	3.15 (0.35) bcd
	*V. paradoxa*	3.01 (0.49) cd	2.10 (0.42) bc	2.59 (0.49) b	4.13 (0.49) d
Two-way ANOVA					
	Season	F = 45.89 *P* < 0.01	F = 51.04 *P* < 0.01	F = 9.66 *P* < 0.01	F = 58.67 *P* < 0.01
	Species	F = 3.61 *P* = 0.03	F = 9.86 *P* < 0.01	F = 13.55 *P* < 0.01	F = 0.26 *P* = 0.85
	Season × Species	F = 2.05 *P* = 0.13	F = 3.25 *P* = 0.04	F = 3.39 *P* = 0.03	F = 1.66 *P* = 0.20

Means in the same columns with different lower-case letters show significant differences between tree/shrub leaves (*p* < 0.05) while numbers in brackets after means of tree/shrub leaves show ± one standard error of the mean.

In Burkina Faso, season and species significantly affected decomposition, and nutrient (N, P and K) release rate constants (Table 6 and Supplementary Table S8; *p* < 0.01). Except for the K release rate, there was a season and species interaction effect on decay constants. Average decomposition rate and N, P, and K release rates in the rainy season were 373%, 166%, 209%, and 174%, respectively, higher (*p* < 0.01) than those in the dry season. *Faidherbia albida* and *P. lucens* decomposition rates in dry season were five-folds lower than in the rainy season. Nitrogen, P and K release rates of *P. lucens* in rainy season were 325%, 567%, and 284%, respectively, higher (*p* < 0.01) than its N, P and K release rates in the dry season. *K. senegalensis* exhibited the lowest decomposition and N, P, and K release rates in both seasons compared with the other species.

**Table 6:** Mineralization rate constants (*k*: % day^− 1^) in the decomposition of leaves of leguminous tree/shrub species (*F. albida* and *P. lucens*) and non-leguminous *K. senegalensis* and *V. paradoxa* after 52 weeks in litterbags placed across season in Saria in the Sudano-Sahelian zone of West Central, in Burkina Faso (rainy, *n* = 5 and dry, *n* = 4).

Season	Species	Mass loss	*N*	*P*	K	
Dry		0.70 (0.26) A	1.02 (0.53) A	1.14 (0.27) A	1.58 (0.84) A	
	*F. albida*	0.77 (0.21) a	0.70 (0.21) a	0.77 (0.28) ab	1.05 (0.35) ab	
	*K. senegalensis*	0.28 (0.07) a	0.28 (0.07) a	0.35 (0.07) a	0.77 (0.28) a	
	*P. lucens*	1.05 (0.56) a	1.12 (0.56) a	1.05 (0.49) ab	1.33 (0.56) ab	
	*V. paradoxa*	0.70 (0.21) a	1.96 (1.26) ab	2.38 (0.24) ab	3.15 (2.17) ab	
		3.31 (0.39) B	1.82 (0.37) B	3.50 (0.39) B	4.32 (0.51) B	
Rainy	*F. albida*	3.71 (0.35) ab	2.80 (0.35) ab	3.71 (0.35) b	4.97 (0.42) b	
	*K. senegalensis*	1.75 (0.21) a	0.91 (0.07) a	1.05 (0.07) ab	2.10 (0.28) ab	
	*P. lucens*	5.04 (0.84) b	1.26 (0.84) b	7.00 (0.91) c	5.11 (0.77) b	
	*V. paradoxa*	2.73 (0.14) ab	2.31 (0.21) ab	2.23 (0.21) ab	5.11 (0.56) b	
Two-way ANOVA						
	Season	F = 90.06 *P* < 0.01	F = 15.40 *P* < 0.01	F = 19.98 *P* < 0.01	F = 18.44 *P* < 0.01	
	Species	F = 11.68 *P* < 0.01	F = 6.77 *P* < 0.01	F = 9.25 *P* < 0.01	F = 3.80 *P* = 0.02	
	Season × Species	F = 4.05 *P* = 0.02	F = 3.44 *P* = 0.03	F = 7.16 *P* = 0.01	F = 1.11 *P* = 0.36	

Means in the same columns with different lower-case letters show significant differences between tree/shrub leaves (*p* < 0.05). Numbers in brackets after tree/shrub leaves of show ± one standard error of the mean.

Among the soil chemical properties (N, P, K, Mg, Ca, and pH), initial leaf chemistry (N, P, K, Mg, Ca, TP, and CT), and climatic data (rainfall, relative humidity and temperature), it was only soil pH, plant leafy K, P, and CT and annual average rainfall that had a significant effect on decomposition rate (Table 7). Whist soil pH and CT of plant leaves negatively (*p* < 0.01; *p* = 0.09, respectively) influenced dry weight decomposition rate, initial tree/shrub leaves K, Mg, and P and annual rainfall positively (*p* = 0.03; *p* = 0.02; *p* = 0.06; *p* < 0.01, respectively) influenced dry weight decomposition rate.

**Table 7:** Effects of initial soil pH, and K, P, Mg, P, and condensed tannin (CT) in the initial plant material, and annual average rainfall on dry weight decomposition rate using a general linear model analysis.

	Estimate	Std. Error	t value	Pr(>|t|)
(Intercept)	8.184***	1.190	6.878	< 0.01
CT	−0.010.	0.006	−1.696	0.096
K	0.061*	0.027	2.232	0.030
Mg	0.177*	0.076	2.330	0.024
P	0.212.	0.109	1.949	0.057
pH	−2.230***	0.211	−10.558	< 0.01
Rainfall	0.001***	0.000	4.009	< 0.01

*Significant at *p* < 0.05, **Significant at *p* < 0.01, ***Significant at *p* < 0.001.

In general, nutrient inputs by leafy biomass varied across species in all three countries, except for K in Senegal, and P during dry season in Burkina Faso. In both Mali and Burkina Faso leafy biomass completely decomposed within 52 weeks after soil application, leading to a complete release of the applied nutrients.

In Senegal, the amounts of N applied from *P. lucens* were 76%, 40%, and 53% higher (*p* < 0.01) than of *G. senegalensis*, *F. albida* and *P. reticulatum*, respectively (Table 8), while the amounts of N remaining at 52 weeks after placement of *P. lucens* were 78%, 14%, and 77% lower (*p* < 0.01) than those of *G. senegalensis*, *F. albida*, and *P. reticulatum*, respectively. The amounts of P applied from *F. albida* were 17%, 32%, and 33% lower (*p* = 0.03) than those of *G. senegalensis*, *P. lucens*, and *P. reticulatum*, respectively. Similarly, the amounts of P remaining 52 weeks after placement of *G. senegalensis* and *P. reticulatum* were 5-folds higher (*p* < 0.01) than in *F. albida* leaves. While released K did not vary between species, the amounts of K remaining in leaves of *F. albida*,* G. senegalensis* and *P. lucens* were 403%, 69%, and 669%, respectively lower (*p* < 0.01) than that of *P.* reticulatum.

**Table 8:** Applied nutrients and nutrients remaining in leaves of leguminous tree/shrub (*F. albida*, *P. lucens* and *P. reticulatum*) and non-leguminous *G. senegalensis* after 52 weeks in litterbags placed during the rainy season in Dahra in the Sahelian zone of Louga, Senegal, (*n* = 5).

Season	Species	Applied nutrients			Nutrient remaining after 52 weeks		
		*N*	*P*	K	*N*	*P*	K
		kg ha^− 1^					
Rainy							
	*F. albida*	125.8 (6.8) b	6.3 (0.1) b	43.8 (4. 6)	22.5 (1.2) c	0.7 (0.0) b	1.6 (0.2) c
	*G. senegalensis*	100.5 (0.5) c	7.6 (0.3) ab	48.2 (0.5)	89.5 (0.4) a	3.6 (0.2) a	4.9 (0.1) b
	*P. lucens*	176.5 (2.7) a	9.3 (0.9) a	66.3 (26.7)	19.3 (0.3) c	0.5 (0.1) b	1.1 (0.4) c
	*P. reticulatum*	115.2 (4.4) b	9.4 (0.9) a	52.4 (2.1)	82.7 (3.2) b	3.3 (0.3) a	8.3 (0.3) a
	F	58.97	4.89	0.13	482.00	78.00	134.50
	P	<0.01	0.03	0.94	<0.01	<0.01	<0.01

Means in the same columns with different lower-case letters show significant differences between tree/shrub leaves (*p* < 0.05). Numbers in brackets after means of tree/shrub leaves show ± one standard error of the mean.

In Mali during the dry season, the amount of N applied by *F. albida leaves* was two-folds greater (*p* < 0.01) than the average from the other tree/shrub leaves (Table 9). In the rainy season, average amounts of N released from *F. albida* and *P. lucens* were 126% larger (*p* < 0.01) than the average of *K. senegalensis* and *V. paradoxa*. Average amounts of P released from the other tree/shrub leaves in dry season, was three-folds greater (*p* = 0.03) than that of *P. lucens*, whereas average amount of P applied from *K. senegalensis* and *V. paradoxa* in the rainy season was 48% lower (*p* = 0.04) than the average from *F. albida* and *P. lucens*. In both, the dry and rainy seasons, the amount of K applied with *K. senegalensis* was 67% and 79%, respectively, greater (*p* < 0.01) than the average amount applied from *F. albida*,* P. lucens*, and *V. paradoxa*.

**Table 9:** Applied nutrients and nutrients remaining from leaves of leguminous tree/shrub (*F. albida* and *P. lucens*) and non-leguminous *K. senegalensis* and *V. paradoxa* after 52 weeks in litterbags placed during the dry and the rainy season in Katibougou in the Sudano-Sahelian zone of Koulikoro, Mali (rainy, *n* = 5 and dry, *n* = 4).

Season	Species	Applied nutrients			Nutrient remaining after 52 weeks			
		*N*	*P*	K	*N*	*P*	K	
		kg ha^− 1^						
Dry		72.8 (4.7) A	5.9 (0.5) A	42.7 (4.0) A				
	*F. albida*	106.5 (8.6) b	7.1 (0.0) ab	33.6 (0.1) a	0	0	0	
	*K. senegalensis*	61.5 (9.3) a	7.5 (2.0) ab	61.1 (14.7) ab	0	0	0	
	*P. lucens*	64.3 (0.8) ab	2.5 (0.0) a	38.9 (0.6) a	0	0	0	
	*V. paradoxa*	58.8 (0.2) a	6.6 (0.1) ab	37.1 (0.4) a	0	0	0	
Rainy		135.2 (7.7) B	9.1 (1.3) B	74.8 (4.7) B				
	*F. albida*	175.4 (21.0) c	1 2.2 (3.1) b	44.9 (11.2) a	0	0	0	
	*K. senegalensis*	83.5 (0.9) ab	6.7 (0.6) ab	111.8 (1.7) c	0	0	0	
	*P. lucens*	199.7 (8.5) c	11.6 (1.0) b	83.3 (3.5) ab	0	0	0	
	*V. paradoxa*	82.3 (0.4) ab	5.7 (0.5) ab	59.3 (2.2) ab	0	0	0	
Two-way ANOVA								
	Season	F = 92.41 *P* < 0.01	F = 10.83 *P* < 0.01	F = 45.55 *P* < 0.01				
	Species	F = 33.64 *P* < 0.01	F = 2.39 *P* = 0.11	F = 18.62 *P* < 0.01				
	Season × Species	F = 16.82 *P* < 0.01	F = 6.66 *P* < 0.01	F = 03.79 *P* = 0.03				

Means in the same columns with different lower-case letters show significant differences between tree/shrub (*p* < 0.05). Numbers in bracket after tree/shrub leaves means show ± one standard error of the mean.

In Burkina Faso, leaf applications of *P. lucens* led to the highest and of *K. senegalensis* to the lowest N application in both seasons (*p* < 0.01) (Table 10). While in the dry season the P application rate was similar for all species, in the rainy season, the amount of P applied from *F. albida*, *K. senegalensis*, and *V. paradoxa* were 83%, 78% and 79%, respectively, lower (*p* < 0.01) than that from *P. lucens*. Whilst the average amounts of K applied in the dry season from *F. albida*, *P. lucens*, and *V. paradoxa* were 140% greater (*p* < 0.01) than that from *K. senegalensis*, in the rainy season, the average amounts of K applied of *K. senegalensis*,* P. lucens*, and *V. paradoxa* was two-folds higher (*p* = 0.02) than that of *F. albida*.

**Table 10:** Applied nutrients and nutrients remaining in leaves of leguminous tree/shrub (*F. albida* and *P. lucens*) and non-leguminous *K. senegalensis* and *V. paradoxa* after 52 weeks in litterbags placed during the dry and the rainy season in Saria in the Sudano-Sahelian zone of West Central, in Burkina Faso (rainy, *n* = 5 and dry, *n* = 4).

Season	Species	Applied nutrients			Nutrient remaining after 52 weeks			
		*N*	*P*	K	*N*	*P*	K	
		kg ha^− 1^						
Dry		65.7 (2.2) A	3.0 (0.4) A	28.3 (2.3) A				
	*F. albida*	74.6 (0.9) ab	3.1 (0.1) a	34.1 (1.5) a	0	0	0	
	*K. senegalensis*	47.3 (0.5) a	2.4 (0.3) a	13.8 (1.3) a	0	0	0	
	*P. lucens*	93.3 (7.1) bc	4.1 (0.7) a	35.7 (0.0) a	0	0	0	
	*V. paradoxa*	47.6 (0.1) a	2.5 (0.6) a	29.7 (6.2) a	0	0	0	
Rainy		112.9 (4.6) B	10.9 (2.1) B	60.25 (5.4) B				
	*F. albida*	111.7 (13.9) c	4.7 (0.4) a	33.4 (3.2) a	0	0	0	
	*K. senegalensis*	80.3 (1.2) b	5.9 (0.6) a	65.9 (2.0) b	0	0	0	
	*P. lucens*	168.3 (2.1) d	27.2 (7.2) b	65.3 (14.1) b	0	0	0	
	*V. paradoxa*	91.3 (1.2) bc	5.7 (0.3) a	76.4 (2.2) b	0	0	0	
Two-way ANOVA								
	Season	F = 141.63 *P* < 0.01	F = 18.62 *P* < 0.01	F = 63.37 *P* < 0.01				
	Species	F = 58.74 *P* < 0.01	F = 10.19 *P* < 0.01	F = 5.01 *P* = 0.01				
	Season × Species	F = 5.75 *P* < 0.01	F = 7.86 *P* < 0.01	F = 8.79 *P* < 0.01				

Means in same columns with different lower-case letters show significant differences between tree/shrub (*p* < 0.05). Numbers in bracket means of after tree/shrub leaves show ± one standard error of the mean.

## Discussion

The findings of our study confirm the hypothesis that nutrient concentrations in tree/shrub leafy biomass undergo seasonal variation. Like other studies in Brazil^20^, China^22^, Nigeria^34^, and India^35^ we observed higher N, P, and K concentrations in leaves of the rainy season than in those of the dry season. High leaf N, P, and K concentrations in the rainy season likely result from an improved uptake of these nutrients from the soil solution due to increased litter decomposition and mineralization^21,35^. In addition, upon the onset of the rainy season, trees and shrubs typically produce new leaves, which contain higher nutrient concentrations than older leaves. Contrary to our findings^19,23,25^ reported higher K concentration in tree leaves of the dry season than of the rainy season from Ethiopia, Nigeria, and Ghana, respectively. An important K source in Sub-Saharan Africa is dust carried from the Sahara desert with the Harmattan winds^8,26^. The fallout of these winds reach regions closer to the Sahara desert, such as the study locations, more than coastal ones and lead to a substantial input of K^36^. In addition, K is not bound to organic compounds and thus highly mobile, making it prone to leaching^37^. The two-fold higher rainfall in the above-mentioned regions compared with our study locations may have led to leaching of K during the rainy season.

Our observation of high total phenol and condensed tannin concentrations of leaves in the dry season confirms findings of other authors^38–40^. As an adaptation response to moisture and nutrient availability, particularly nitrogen N, trees tend to increase condensed tannin production to protect leaf resources from herbivory^39^ but also from fungal attack. Hence, trees’ preferential allocation of C for leaf defence instead for growth leads to higher tannin concentrations in the dry season. Additionally, trees exposed to high solar radiation and under nutrient stress, tend to increase phenolics production to prevent photodamage from excess radiation^41^. Irrespective of location and season average N, P, and Mg concentrations in leguminous tree/shrub leaves of *F. albida*,* P. lucens*, and *P. reticulatum* were significantly higher than in non-leguminous tree/shrub leaves (*G. senegalensis*, *K. senegalensis*, and *V. paradoxa*). Average N concentration in leguminous tree/shrub leaves (22 mg g^− 1^) is comparable with the reported concentration of 20 mg g^− 1^ in the Sudanian savanna of Ghana^42^. Average P concentration of leguminous species (1.5 mg g^− 1^) fall within the range (1.4 to 1.6) reported by ^23^ from Ethiopia. Similarly, our average Mg concentration recorded in leguminous leaves of 3.6 mg g^− 1^ is in the range of 3.4 mg g^− 1^ to 6.7 mg g^− 1^ reported by ^29^ from Ghana. Leguminous tree/shrub species accumulate higher N concentration through their association with N_2_-fixing bacteria^43^. Because legumes require high amounts of P for N_2_ fixation^44^ and Mg to support host legume and rhizobia growth^45^, legumes associate with arbuscular mycorrhiza which are known to be more efficient at scavenging for these nutrients^44^. Our data show that non-legumes accumulated more total phenols and condensed tannins than legumes, which confirmed earlier observations^46^,^47^. Given their protein binding properties condensed tannins are a powerful plant defence against predators and herbivores^39^ by reducing leaf palatability and digestibility^48^. Under N limiting conditions, as it was likely the case for non-leguminous tree/shrub species at the study locations, plants allocate more C to secondary compounds such as condensed tannins, whereby C accumulates in excess of growth demands^49^.

In both Burkina Faso and Mali seasonality had no effect on N concentration of *F. albida*, however, for *P. lucens*, N concentration in the dry season was 35% lower than in the rainy season. Contrary to *P. lucens* which sheds its leaves in the dry season^50^, *F. albida* maintains leaves throughout the dry season^51^. Consequently, reduced transfer of C from leaves of *P. luces* to rhizobia in the dry season may cause limited N_2_ fixation and ultimately reduced N concentration in the leaves during the dry season, whereas *F. albida* was able to maintain its N_2_ fixing capacity during both seasons.

Across locations and seasons, the average decomposition rate of 0.23 *k* week^− 1^ was between the 0.02 to 0.41 *k* week^− 1^ reported for decomposition rates of tree/shrub leaves in Burkina Faso and Nigeria^11,52,53^. The initial rapid decomposition found in our study across countries and seasons, may be attributed to the break down and leaching of soluble components such as sugar, starch, and amino acids as earlier reported^25,54^. The rate of K release was 55% faster than that of N and 18% faster than that of P. It was also 42% faster than DM losses. These observations support earlier findings^55^,^47^ reflecting easy leaching of this highly soluble nutrient^55,56^. Seasonality affected decomposition and nutrient release rates from leaves, with the rainy season leading to 155%, 97%, 112%, and 115% higher decomposition rates and N, P, and K release rates, respectively, than the dry season. Similar findings have been reported by from Ethiopia^57^ and Brazil^20^. The decomposition rate is influenced by the quality of the plant residues: high quality residues with low C: N ratios decompose faster in moist conditions^58^. The effects of rainfall on decomposition of plant residue are attributable to rainfall-enhanced moisture availability. Moisture availability is crucial for microbial activity and nutrient release, supporting its positive effect on decomposition rate in our study. Thus the fast decomposition and nutrient release during the rainy season was most likely caused by higher soil moisture, which promotes soil fauna activity and enzymatic processes^18,20^. Rainfall promoting litter decomposition and nutrient release have been reported in earlier studies from Costa Rica^56^ and Eastern Africa^59^. Aside soil moisture, the combination between warm temperature and high humidity in the rainy season is well known to hasten microbial activity which increases decomposition and nutrient release during the rainy season^16,22^. While there was no significant differences between average temperature between dry and rainy season at any of our study locations, average relative humidity in the rainy season was 97% higher than in the dry season. In contrast to our findings,^14^ noted that *Albizia gummifera *G. F. Gmel. leaves decompose 3% faster in the dry season than in the rainy season of Ethiopia. The disparity between our finding and that of the above author is attributed to seasonal differences between study sites. Compared with our study locations which do not experience rains during the dry season,^14^ stated that at their study location sporadic rainfalls occurred in the dry season which increased decomposition under high temperature.

The average decomposition rate of leafy biomass of leguminous tree/shrub was 1.4-fold higher than of non-leguminous tree/shrub leaves confirming earlier findings. The compared with their non-leguminous counterparts higher initial leaf N, P, and Mg concentrations in leguminous tree/shrub leaves were found to be positively correlated with decomposition^60,61^. Contrarily, non-leguminous tree/shrub trees had higher C/N ratios and larger concentrations of total phenols and condensed tannins, which have been reported to negatively affect decomposition^62,63^. A low C/N ratio facilitates the breakdown of organic matter whereby C/N ratios optimal for decomposition range from 20 to 30^64^. In our study non-leguminous leaves had a C/N ratio of 37 compared with 22 in legume species, resulting in their slower decomposition even though no statistically significant influence of the C/N ratio was found in our overall analysis of mineralization determinants. The positive effect of P on leaf decomposition in our study supports earlier work that acknowledged its pivotal role in litter decomposition of terrestrial ecosystems^65–67^, Similar results on matter decomposition have been reported from northern Arizona^68^ and China^65^. Condensed tannins are essential determinants of decomposition^32^ because of their antimicrobial activity and high reactivity with N and related compounds. High condensed tannin levels in initial leaves have been reported to slowdown decomposition rates^32,33^.

## Materials and methods

### Study area

The study was conducted at Dahra (15.2410º N, 15.2556º W) in the Sahelian ecological zone of Senegal and at Koulikoro (17.5707° N, 3.9962° W) and Saria (12.2383° N, 1.5616° W) in the Sudano-Sahelian ecological zones of Mali and Burkina Faso, respectively. In 2021 annual rainfall in Dahra was 420 mm with 74% (309 mm) of rainfall occurring in August. In Dahra, average annual temperature was 29 °C of which the highest temperature of 32 °C was recorded in April and June, and the lowest temperature of 25 °C in January. For Koulikoro, average annual rainfall was 705 mm in 2021 with July having the highest rainfall (233 mm corresponded to 33% of the total annual rainfall). The annual average temperature was 28 °C with a maximum of 33 °C occurring the April, and May a minimum of 24 °C occurring in December and January. From 2021 to 2022 in Saria, the 2-year average rainfall was 980 mm with 230 mm occurring in August, and annual temperature averaging 28 °C with a monthly high of 31 °C occurring in March, April, and May and an average low of 25 °C in December (Fané, unpublished data). Soils at the study locations of Mali and Burkina Faso are classified as Ferrasols^69^, and Arenosols for Senegal^70^, with pH ranging from 4.93 to 6.15 and major elements ranging from 2.92 to 4.53 mg C g^− 1^ soil, 0.26 to 0.42 mg N g^− 1^ soil, 0.17–0.21 mg P g^− 1^ soil, 0.05–0.41 mg K g^− 1^, 0.16–0.24 mg Ca g^− 1^ soil, and 0.16–0.23 mg Mg g^− 1^ soil (Fané, unpublished data; Table 11). At all study locations, the vegetation consists of open parklands with continuous grasses and scattered trees^71–73^.

**Table 11:** Top soil (0–20) chemical properties for Dahra in Senegal, Koulikoro in Mali, and Saria in Burkina Faso (*n* = 3).

Study sites	C	*N*	*P*	K	Ca	Mg	Al	Fe	Cr	pH
			mg g^− 1^							
Senegal	2.92 (0.02)	0.26 (0.03)	0.16 (0.00)	0.05 (0.01)	0.16 (0.00)	0.16 (0.00)	3.13 (0.00)	2.60 (0.00)	0.01 (0.00)	6.15 (0.01)
Mali	3.46 (0.01)	0.28 (0.01)	0.17 (0.00)	0.33 (0.01)	0.24 (0.00)	0.21 (0.00)	3.41 (0.00)	2.60 (0.00)	0.04 (0.00)	5.28 (0.05)
Burkina Faso	4.53 (0.03)	0.42 (0.01)	0.21 (0.00)	0.41 (0.01)	0.23 (0.00)	0.23 (0.00)	3.41 (0.00)	2.61 (0.00)	0.04 (0.01)	4.93 (0.02)

Numbers in bracket below means show ± one standard error of the mean.

### Data collection

At each location the decomposition of leafy biomass of four different plant species were investigated: in Mali and Burkina Faso of the non-legume species *V. paradoxa*,* K. senegalensis* with the legume species *F. albida*,* Pterocarpus lucens* Lepr., and in Senegal with the legume species *F. albida*,* P. lucens*,* P. reticulatum* and the non-legume species *G. senegalensis*. Senegal’s species composition differed from that of Mali and Burkina Faso because of the absence of natural stands of *V. paradoxa* and *K. senegalensis* in Dahra (Senegal). In Mali and Burkina Faso decomposition and nutrients release patterns of the plant leaves were studied using the litterbag technique with 20 cm × 20 cm nylon bags with a mesh size of of 2 mm in the dry (from January 2022 to February 2023) and rainy (from June 2021 to July 2022) seasons. In Senegal, the study was conducted only during the rainy season because of limited availability of plant material during the dry season. Each litterbag was filled with fresh leaves equivalent to 26 g DM for the rainy season and 17 g DM for the dry season. In each country per season a total of 120 litterbags were buried at the depth of 15 cm (plough depth) with a 50 cm spacing between the litterbags and arranged in a complete randomized design in five replicates for the rainy and four replications for the dry season. The litterbags were collected 2, 4, 8, 16, 32, and 52 weeks after placement in five and four respective replicates for the rainy and dry seasons. Leaves remaining in the litterbag at each sampling time were carefully separated from soil and organic debris using a brush, oven dried at 60 °C for 72 h, weighed, and milled.

### Chemical analysis

Carbon (C) and nitrogen (N) concentration in the milled samples were analysed by high-temperature combustion using a Vario Max CHN analyzer (Elementar Analysensysteme GmbH, Hanau, Germany). For the determination of phosphorus (P), potassium (K), and magnesium (Mg) concentrations milled samples were ashed at 550 °C for 24 h. The remaining ash was digested with 32% HCl to remove soil particles. The nutrients in the ashing solution were measured by inductively coupled plasma optical emission spectroscopy (Spectrogreen ICP-OES, SPECTRO Analytical Instruments GmbH, Kleve, Germany). Total phenol contents were determined using the Folin-Ciocalteu method^74,75^ in aqueous acetone (70% v/v) as extractant^76^. Total tannins were analyzed by subtracting non-tannin phenols (NTP) after precipitating tannins with polyvinyl polypyrrolidone from total phenols (TP). Condensed tannins (CT) were assessed using the butanol-HCl method, with a 95:5 v/v ratio of butanol to HCl^76^.

### Data analysis

Mineral soil contamination of samples was corrected by using the ash content as an indicator as it performed statistically better than chromium (Cr), iron (Fe) and aluminium (Al) contents (Supplementary Tables S3, S4, S5). To this end the equation 77 as described by^25^ was used:

1 $$\:DM_{{LB}} \left( g \right) \times C_{{LB}} \left( {\frac{{mg}}{g}} \right) = \:DM_{S} \left( g \right) \times C_{S} \left( {\frac{{mg}}{g}} \right) + \:DM_{L} \left( g \right) \times C_{L} \left( {\frac{{mg}}{g}} \right)$$

2 $$\:DM_{{LB}} \left( g \right) = \:DM_{S} \left( g \right) + \:DM_{L} \left( g \right)$$

The two equations were combined, resulting in Eq. 3, which was used to estimate the contamination of litterbag content by soil particles.

3 $$\:DM_{S} \left( g \right)\: = \:\frac{{DM_{{LB}} \left( g \right) \times \left[ {C_{{LB}} \left( {\frac{{mg}}{g}} \right)\: - C_{L} \left( {\frac{{mg}}{g}} \right)} \right]}}{{C_{S} \left( {\frac{{mg}}{g}} \right)\: - C_{L} \left( {\frac{{mg}}{g}} \right)}}$$

where *DM*_*S*_ *(g)* is the soil DM (actual contamination), *DM*_*LB*_ *(g)* is the litterbag DM, $\:C_{{LB}} \left( {\frac{{mg}}{g}} \right)$ is the ash concentration in litterbags, $\:C_{S} \left( {\frac{{mg}}{g}} \right)$ is the ash concentration in the soil, and $\:C_{L} \left( {\frac{{mg}}{g}} \right)$ is the ash concentration in litter/leaves.

The percent dry weight or nutrient remaining in the litterbag at each sampling time was determined using the following equation:

4 $$\:DM_{{r\:}} \left( \% \right) = \left( {\frac{{DM_{{t\:}} }}{{DM_{0} }}} \right) \times 100$$

where DMr is the % dry matter or nutrient quantity of leaves remaining in the litterbag at each sampling time, DM_t_ is the dry matter or nutrient content of leaves remaining in the litterbag at each sampling time, and DW_0_ is the initial dry weight or nutrient content of the leaves.

Rates of decomposition and nutrient release were estimated with Sigmaplot 15 (Systat Software Inc., San Jose, CA, USA) using the two-parameter exponential model proposed^78^: $\:Y = {\text{ }}\beta _{I} e^{{ - kt}}$, where Y is the remaining material at sampling time t in percent of the initial material, *β*_*i*_ is the recalcitrant pool fraction of species i and k is the decay or nutrient release rate.

T-tests were used to compare initial nutrient concentration, decomposition and nutrient release rates between species types (legumes and non-legumes). One-way analysis of variance (ANOVA) was employed to compare plant leaves’ initial nutrient concentration, and their decomposition and nutrient release rates in Senegal. In Mali and Burkina Faso, two-way ANOVA was used to test the interaction between different plant leaves and seasonality on leaves’ initial nutrient concentrations, and their decomposition and nutrient release rates. Data violating the assumptions of ANOVA were transformed using the Tukey ladder of powers. The generalised linear model assuming a Gaussian error distribution was used to determine the soil (N, P, K, Mg, Ca and pH), and initial leaves chemical (N, P, K, Mg, Ca, TP and CT) properties and climate data (rainfall, relative humidity and temperature) affecting the decomposition rate of tree/shrub leaves at the study locations. To this end, non-significant variables were sequentially removed from the model by eliminating the one with the highest *p* value until all remaining variables were significant. Prior to fitting the model, rates of leaf decomposition were log transformed to satisfy the linearity assumption of the model. All statistics were performed using the R statistical software version 4.3.3^79^.

## Conclusions

The results of this study showed that season significantly influenced chemical properties of the evaluated leguminous tree/shrub species. The decomposition rates and nutrient release patterns across locations were higher during the rainy season especially for leaves of species with higher concentrations of N, P, and K compared with observations during the dry season. In contrast, C/N ratio, total phenol and condensed tannin concentrations of leaves were higher in the dry than in the rainy season. Results also showed that the leaves of tree/shrub species differed in chemical properties, whereby leguminous tree/shrub leaves recorded higher N, P and Mg concentrations than non-leguminous species. The leaves of non-leguminous tree/shrub on the other hand, recorded higher concentrations of total phenols, K, and condensed tannins. Decomposition rates of leguminous tree/shrub leafy biomass were higher than of non-leguminous tree/shrub leaves which is attributable to the generally higher nutrient concentrations and lower total phenol concentrations in legumes.

To improve their application efficiency at the farm level, leaf mulching should be tailored according to the species type and rate of biomass application for a particular environment. This will help to optimize synchronization of nutrient release with crop nutrient demands. Overall, the leaves of leguminous tree/shrub species seem better suited for use as organic fertilizers to meet early nutrient demands by crops, whereas non-leguminous tree/shrub leaves appear more suitable for use as long-term soil conditioners and slow-release organic fertilizers.

## Supplementary Information

Below is the link to the electronic supplementary material.

Supplementary Material 1

## Data Availability

Data is provided within the manuscript and original data can be obtained from the corresponding author upon reasonable request.

## Declarations

### Plant material identification

The species studied were identified and authenticated by Dr Sidi Sanogo, researcher, and curator of the herbarium (http://sciweb.nybg.org/science2/indexherbariorum.asp) at the Institut d’Economie Rurale (IER) du Mali and a co-author of the manuscript. Samples of seeds and/or herbariums observed: *Faidherbia albida* (129-UPP000), *Piliostigma reticulatum* (227-UPP-068), *Pterocarpus lucens* (237-UPP-072), *Khaya senegalensis* (169-UPP-029-1) and *Guiera senegalensis* (ML-USFH-79).
