# Supplementary material for: Decomposition and nutrient release from leaves of some common agroforestry tree/shrub species of Sudano-Sahelian West Africa
Source: Sci Rep. 2026 Jan 24;16:3273. doi: 10.1038/s41598-025-29117-9 (PMC12835154; doi:10.1038/s41598-025-29117-9)
Supplement: Supplementary file 1 — Supplementary Material 1 [file 41598_2025_29117_MOESM1_ESM.docx]

**Decomposition and nutrient release from leaves of some common agroforestry tree/shrub species of Sudano-Sahelian West Africa**

**Supplementary materials**

***Initial plant chemical properties***

**Table S1.** Initial chemical properties of leaves of leguminous trees/shrubs (*F. albida, P. reticulatum* and *P. lucens*) and non-leguminous species (*K. senegalensis, G. senegalensis* and *V. paradoxa*) subjected to a decomposition study during the rainy season in Dahra, Sahelian zone of Senegal, Katibougou, and Saria, Sudano-Sahelian zone of Koulikoro in Mali and West Central in Burkina Faso, respectively (n = 3)

| Countries | Species | N | P | K | Mg | C:N | Total Phenols | Condensed-Tannin |
| --- | --- | --- | --- | --- | --- | --- | --- | --- |
|  |  | mg g^-1^ | | | | | | |
| Senegal |  | 19.92 (1.36) A | 1.25 (0.07) A | 8.10 (0.97) A | 3.58 (0.51) A | 24.85 (1.50) A | 34.72 (2.01) A | 23.83 (5.09) A |
|  | Leguminous | 21.41 (1.51) b | 1.28 (0.10) ab | 8.33 (1.31) a | 3.85 (0.66) a | 22.70 (1.34) b | 32.16 (2.01) a | 25.61 (7.97) a |
|  | Nonleguminous | 15.46 (0.08) ab | 1.17 (0.06) ab | 7.41 (0.07) a | 2.80 (0.05) a | 31.31 (0.12) c | 42.41 (1.08) ab | 18.51 (1.36) a |
| Mali |  | 20.80 (2.57) A | 1.39 (0.17) A | 11.51 (1.24) A | 3.94 (0.33) A | 27.10 (3.22) B | 47.69 (10.06) A | 30.23 (0.03) A |
|  | Leguminous | 28.85 (1.77) c | 1.83 (0.22) ab | 9.86 (1.55) a | 4.51 (0.32) a | 16.56 (0.94) a | 27.58 (0.43) a | 28.68 (6.12) a |
|  | Nonleguminous | 12.76 (0.08) a | 0.95 (0.05) a | 13.16 (1.82) a | 3.34 (0.49) a | 37.63 (0.53) c | 67.78 (16.85) ab | 31.78 (1.44) a |
| Burkina Faso |  | 17.37 (1.64) A | 1.67 (0.50) A | 9.27 (0.89) A | 3.42 (0.24) A | 29.70 (2.38) C | 52.60 (1213) A | 32.29 (5.59) A |
|  | Leguminous | 21.54 (2.18) b | 2.45 (0.92) b | 7.59 (1.48) a | 4.01 (0.32) a | 22.79 (2.31) b | 24.62 (3.31) a | 34.93 (10.93) a |
|  | Nonleguminous | 13.21 (0.39) a | 0.87 (0.10) a | 10.95 (0.42) a | 2.82 (0.14) a | 36.61 (1.34) c | 80.59 (17.98) | 29.65 (3.92) a |
| Two-way ANOVA | | | | | | | | |
|  | Countries | F = 2.84  *P* = 0.07 | F = 10.01  *P* = 0.23 | F = 2.96  *P* = 0.07 | F = 0.54  *P* = 0.59 | F = 6.31  *P* < 0.01 | F = 1.63  P = 0.21 | F = 0.71  P = 0.50 |
|  | Types | F = 67.67  *P* < 0.01 | F = 7.60  *P* < 0.01 | F = 3.20  *P* = 0.08 | F = 7.11  *P* = 0.01 | F = 166.33  *P* < 0.01 | F = 18.74  P < 0.01 | F = 0.19  P = 0.67 |
|  | Countries × Type | F = 5.75  *P* < 0.01 | F = 1.46  *P* = 0.03 | F = 1.22  *P* = 0.31 | F = 0.01  *P* = 0.99 | F = 9.21  *P* < 0.01 | F = 2.16  P < 0.01 | F = 0.25  P = 0.78 |

Means in the same columns with different lower-case letters show significant differences between plant types and upper-case letters show significant differences between countries (p < 0.05). Numbers in brackets after means of plant types show ± one standard error of the mean.

**Table S2.** Initial chemical properties of leaves of leguminous trees/shrubs (*F. albida* and *P. lucens*) and non-leguminous species (*K. senegalensis* and *V. paradoxa*) subjected to a decomposition study across seasons in Katibougou, and Saria, Sudano-Sahelian zone of Koulikoro in Mali and West Central in Burkina Faso, respectively (Rainy and Dry, n = 3)

| Countries | Season | N | P | K | Mg | C:N | Total Phenols | Condensed-Tannin |
| --- | --- | --- | --- | --- | --- | --- | --- | --- |
|  |  | mg g^-1^ | | | | | | |
| Mali |  | 18.69 (1.54) A | 1.36 (0.27) A | 10.51 (0.74) A | 3.81 (0.20) A | 28.75 (1.87) A | 59.93 (6.44) A | 36.42 (3.17) A |
|  | Dry | 16.58 (1.57) a | 1.32 (0.15) a | 9.51 (0.78) ab | 3.68 (0.27) ab | 30.41 (1.94) a | 72.18 (6.67) ab | 42.62 (5.09) a |
|  | Rainy | 20.80 (2.57) a | 1.39 (0.17) a | 11.51 (1.24) b | 4.02 (0.33) b | 27.10 (3.22) a | 47.69 (10.06) a | 30.23 (0.03) a |
| Burkina Faso |  | 16.41 (1.54) A | 1.20 (0.27) A | 7.95 (0.74) B | 3.09 (0.19) B | 31.58 (1.86) A | 70.40 (7.61) A | 38.51 (3.67) A |
|  | Dry | 15.45 (1.42) a | 0.71 (0.07) a | 6.41 (0.69) a | 2.76 (0.26) a | 33.45 (2.86) a | 88.19 (6.13) b | 44.73 (4.26) a |
|  | Rainy | 17.37 (1.64) a | 1.67 (0.50) a | 9.27 (0.89) ab | 3.42 (0.24) ab | 29.70 (2.38) b | 52.60 (12.13) a | 32.29 (5.59) a |
| Two-way ANOVA | | | | | | | | |
|  | Countries | F = 1.52  *P* = 0.22 | F = 0.37  *P* = 0.55 | F = 7.69  *P* < 0.01 | F = 0.54  *P* = 0.59 | F = 1.14  *P* = 0.29 | F = 1.32  P = 0.26 | F = 0.21  P = 0.65 |
|  | Season | F = 2.74  *P* = 0.10 | F = 3.46  *P* = 0.07 | F = 6.31  *P* = 0.02 | F = 7.11  *P* = 0.01 | F = 1.78  *P* = 0.19 | F = 10.92  P < 0.01 | F = 7.29  P = 0.01 |
|  | Countries × Season | F = 0.35  *P* = 0.54 | F = 2.63  *P* = 0.11 | F = 0.12  *P* = 0.74 | F = 0.01  *P* = 0.99 | F = 0.01  *P* = 0.94 | F = 0.37  P = 0.55 | F = 0.00  P = 1.00 |

Means in the same columns with different lower-case letters show significant differences between seasons and upper-case letters show significant differences between countries (p < 0.05). Numbers in brackets after season means show ± one standard error of the mean.

***Soil contamination correction factors***

**Table S3.** Nonlinear regression model for relative dry weight correction factors (Ae ^̶ kt^) of leaves after 52 weeks in litterbags placed on the soil during the rainy season in Dahra in the Sahelian zone of Senegal, Katibougou and Saria in the Sudano-Sahelian of Mali and Burkina Faso, respectively. A stands for the labile fraction, k for the decay or nutrient release rate and t_50_ is the half-life in weeks. R² denotes the goodness of fit of the decay curve.

| Season | Factors/species | A | K | t_50_ | R² | *P*-value |
| --- | --- | --- | --- | --- | --- | --- |
| Rainy | Ash correction | | | | | |
|  | *F. albida* | 76.26 | 0.03 | 22.15 | 0.78 | <0.0001 |
|  | *G. senegalensis* | 83.74 | 0.02 | 34.66 | 0.84 | <0.0001 |
|  | *P. lucens* | 81.61 | 0.03 | 25.21 | 0.88 | <0.0001 |
|  | *P. reticulatum* | 79.17 | 0.02 | 34.48 | 0.72 | <0.0001 |
|  | Cr correction | | | | | |
|  | *F. albida* | 92.84 | 0.35 | 2.00 | 0.37 | <0.0001 |
|  | *G. senegalensis* | 71.48 | 0.04 | 19.80 | 0.51 | <0.0001 |
|  | *P. lucens* | 119.13 | 0.18 | 3.93 | 0.79 | <0.0001 |
|  | *P. reticulatum* | 85.33 | 0.08 | 9.17 | 0.61 | <0.0001 |
|  | Fe correction | | | | | |
|  | *F. albida* | 98.61 | 0.46 | 1.52 | 0.66 | <0.0001 |
|  | *G. senegalensis* | 704.18 | 0.00 | 294993905843.28 | -0.03 | <0.0001 |
|  | *P. lucens* | 111.09 | 0.25 | 2.74 | 0.91 | <0.0001 |
|  | *P. reticulatum* | 107.47 | 0.27 | 2.53 | 0.91 | <0.0001 |
|  | Al correction | | | | | |
|  | *F. albida* | 94.47 | 0.25 | 2.73 | 0.62 | <0.0001 |
|  | *G. senegalensis* | 819.50 | 0.00 | 6503538943.14 | -0.04 | <0.0001 |
|  | *P. lucens* | 108.79 | 0.21 | 3.26 | 0.90 | <0.0001 |
|  | *P. reticulatum* | 101.54 | 0.19 | 3.65 | 0.90 | <0.0001 |

Lowercase letters indicate significant differences between plant species (n = 4) across seasons (n = 2) at *P* < 0.05.

**Table S4.** Nonlinear regression model for relative dry weight correction factors (Ae ^̶ kt^) of leaves after 52 weeks in litterbags placed on the soil during the rainy and dry season in Katibougou in the Sudano-Sahelian of Koulikoro in Mali. A stands for the labile fraction, k for the decay or nutrient release rate and t_50_ is the half-life in weeks. R² denotes the goodness of fit of the decay curve.

| Seasons | Factors/species | A | | | K | | t_50_ | | R² | | *P*-value |
| --- | --- | --- | --- | --- | --- | --- | --- | --- | --- | --- | --- |
| Rainy | Ash correction | | | | | | | | | | |
|  | *F. albida* | 94.65 | | | 0.54 | | 1.27 | | 0.88 | | <0.0001 |
|  | *K. senegalensis* | 78.05 | | | 0.23 | | 3.07 | | 0.83 | | <0.0001 |
|  | *P. lucens* | 87.10 | | | 0.36 | | 1.95 | | 0.88 | | <0.0001 |
|  | *V. paradoxa* | 85.94 | | | 0.34 | | 2.02 | | 0.82 | | <0.0001 |
|  | Cr correction | | | | | | | | | | |
|  | *F. albida* | 96.13 | | | 0.49 | | 1.40 | | 0.95 | | <0.0001 |
|  | *K. senegalensis* | 86.86 | | | 0.20 | | 3.51 | | 0.90 | | <0.0001 |
|  | *P. lucens* | 87.18 | | | 0.30 | | 2.32 | | 0.89 | | <0.0001 |
|  | *V. paradoxa* | 84.80 | | | 0.37 | | 1.87 | | 0.79 | | <0.0001 |
|  | Fe correction | | | | | | | | | | |
|  | *F. albida* | 100.42 | | | 0.64 | | 1.07 | | 0.94 | | <0.0001 |
|  | *K. senegalensis* | 88.35 | | | 0.37 | | 1.86 | | 0.83 | | <0.0001 |
|  | *P. lucens* | 96.19 | | | 0.45 | | 1.54 | | 0.93 | | <0.0001 |
|  | *V. paradoxa* | 52.75 | | | 0.19 | | 3.64 | | 0.05 | | 0.1851 |
|  | Al correction | | | | | | | | | | |
|  | *F. albida* | 100.19 | | | 0.54 | | 1.29 | | 0.97 | | <0.0001 |
|  | *K. senegalensis* | 88.05 | | | 0.30 | | 2.28 | | 0.87 | | <0.0001 |
|  | *P. lucens* | 95.01 | | | 0.43 | | 1.60 | | 0.92 | | <0.0001 |
|  | *V. paradoxa* | 53.10 | | | 0.20 | | 3.41 | | 0.06 | | 1.3894 |
| Dry | Ash correction | | | | | | | | | | |
|  | *F. albida* | | 101.11 | 0.12 | | 5.61 | | 0.77 | | <0.0001 | |
|  | *K. senegalensis* | | 106.74 | 0.05 | | 12.98 | | 0.68 | | <0.0001 | |
|  | *P. lucens* | | 87.99 | 0.10 | | 6.82 | | 0.59 | | <0.0001 | |
|  | *V. paradoxa* | | 85.31 | 0.08 | | 8.98 | | 0.58 | | <0.0001 | |
|  | Cr correction | | | | | | | | | | |
|  | *F. albida* | | 98.39 | 0.14 | | 5.13 | | 0.80 | | <0.0001 | |
|  | *K. senegalensis* | | 103.18 | 0.06 | | 12.27 | | 0.70 | | <0.0001 | |
|  | *P. lucens* | | 86.11 | 0.13 | | 5.42 | | 0.49 | | 0.0001 | |
|  | *V. paradoxa* | | 85.81 | 0.09 | | 7.33 | | 0.54 | | 0.0001 | |
|  | Fe correction | | | | | | | | | | |
|  | *F. albida* | | 97.51 | 0.45 | | 1.55 | | 0.86 | | <0.0001 | |
|  | *K. senegalensis* | | 93.14 | 0.11 | | 6.32 | | 0.71 | | <0.0001 | |
|  | *P. lucens* | | 104.11 | 0.34 | | 2.04 | | 0.77 | | <0.0001 | |
|  | *V. paradoxa* | | 83.88 | 0.11 | | 6.18 | | 0.57 | | <0.0001 | |
|  | Al correction | | | | | | | | | | |
|  | *F. albida* | | 96.30 | 0.48 | | 1.45 | | 0.83 | | <0.0001 | |
|  | *K. senegalensis* | | 107.51 | 0.11 | | 6.16 | | 0.76 | | <0.0001 | |
|  | *P. lucens* | | 104.82 | 0.42 | | 1.66 | | 0.89 | | <0.0001 | |
|  | *V. paradoxa* | | 83.18 | 0.11 | | 6.33 | | 0.56 | | 0.0001 | |

Lowercase letters indicate significant differences between plant species (n = 4) across seasons (n = 2) at *P* < 0.05.

**Table S5.** Nonlinear regression model for relative dry weight correction factors (Ae ^̶ kt^) of leaves after 52 weeks in litterbags placed on the soil during the rainy and dry season in Saria in the Sudano-Sahelian of West Central in Burkina Faso. A stands for the labile fraction, k for the decay or nutrient release rate and t_50_ is the half-life in weeks. R² denotes the goodness of fit of the decay curve.

| Seasons | Factors/species | | | B | K | t_50_ | R² | *P*-value |
| --- | --- | --- | --- | --- | --- | --- | --- | --- |
| Rainy | | Ash correction | | | | | | |
|  |  | *F. albida* | 95.42 | | 0.49 | 1.41 | 0.88 | <0.0001 |
|  |  | *K. senegalensis* | 83.48 | | 0.20 | 3.55 | 0.89 | <0.0001 |
|  |  | *P. lucens* | 98.68 | | 0.75 | 0.92 | 0.90 | <0.0001 |
|  |  | *V. paradoxa* | 87.06 | | 0.33 | 2.09 | 0.84 | <0.0001 |
|  |  | Cr correction | | | | | | |
|  |  | *F. albida* | 101.06 | | 0.46 | 1.49 | 0.86 | <0.0001 |
|  |  | *K. senegalensis* | 96.92 | | 0.29 | 2.39 | 0.92 | <0.0001 |
|  |  | *P. lucens* | 99.20 | | 0.63 | 1.10 | 0.85 | <0.0001 |
|  |  | *V. paradoxa* | 77.24 | | 0.28 | 2.44 | 0.57 | <0.0001 |
|  |  | Fe correction | | | | | | |
|  |  | *F. albida* | 100.56 | | 0.76 | 0.91 | 0.88 | <0.0001 |
|  |  | *K. senegalensis* | 98.19 | | 0.40 | 1.72 | 0.55 | <0.0001 |
|  |  | *P. lucens* | 100.80 | | 0.79 | 0.88 | 0.88 | <0.0001 |
|  |  | *V. paradoxa* | 100.00 | | 7.17 | 0.10 | 0.80 | <0.0001 |
|  |  | Al correction | | | | | | |
|  |  | *F. albida* | 102.00 | | 0.59 | 1.17 | 0.78 | <0.0001 |
|  |  | *K. senegalensis* | 40.33 | | 0.02 | 37.27 | 0.12 | <0.0001 |
|  |  | *P. lucens* | 100.69 | | 0.76 | 0.91 | 0.83 | <0.0001 |
|  |  | *V. paradoxa* | 100.00 | | 2.72 | 0.25 | 0.48 | <0.0001 |
| Dry | Ash correction | | | | | | | |
|  | *F. albida* | | | 94.60 | 0.11 | 6.48 | 0.58 | <0.0001 |
|  | *K. senegalensis* | | | 102.54 | 0.05 | 14.59 | 0.63 | <0.0001 |
|  | *P. lucens* | | | 107.68 | 0.12 | 6.02 | 0.67 | <0.0001 |
|  | *V. paradoxa* | | | 90.63 | 0.08 | 8.54 | 0.32 | 0.0004 |
|  | Cr correct | | | | | | | |
|  | *F. albida* | | | 86.57 | 0.12 | 5.95 | 0.53 | <0.0001 |
|  | *K. senegalensis* | | | 100.09 | 0.05 | 14.84 | 0.61 | <0.0001 |
|  | *P. lucens* | | | 106.71 | 0.12 | 5.92 | 0.68 | <0.0001 |
|  | *V. paradoxa* | | | 87.49 | 0.08 | 8.41 | 0.33 | 0.0004 |
|  | Fe correction | | | | | | | |
|  | *F. albida* | | | 100.00 | 2.20 | 0.32 | 0.88 | <0.0001 |
|  | *K. senegalensis* | | | 97.72 | 0.27 | 2.60 | 0.33 | 0.0005 |
|  | *P. lucens* | | | 101.55 | 0.25 | 2.74 | 0.77 | <0.0001 |
|  | *V. paradoxa* | | | 65.92 | 0.11 | 6.21 | 0.29 | 0.0014 |
|  | Al correction | | | | | | | |
|  | *F. albida* | | | 100.00 | 6.86 | 0.10 | 0.77 | <0.0001 |
|  | *K. senegalensis* | | | 106.35 | 0.49 | 1.42 | 0.13 | 0.0036 |
|  | *P. lucens* | | | 94.90 | 0.27 | 2.56 | 0.70 | 0.0029 |
|  | *V. paradoxa* | | | 72.53 | 0.25 | 2.75 | 0.26 | 0.0049 |

Lowercase letters indicate significant differences between plant species (n = 4) across seasons (n = 2) at *P* < 0.05.

***Decomposition and nutrient release of leaves***

**
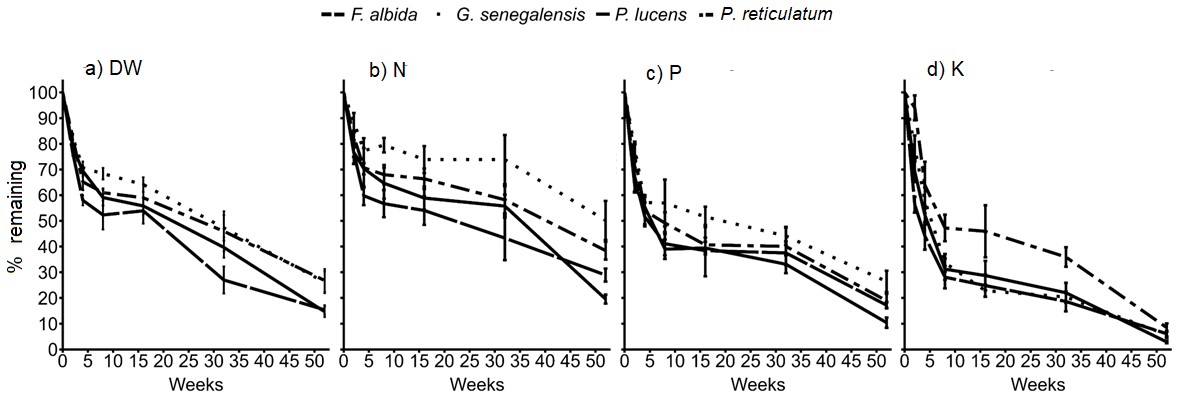
**

**Figure S1.** Decay and nutrient release patterns of different tree/shrub leaves after 52 weeks in litterbags placed on the soil during the rainy season in Dahra in the Sahelian zone of Senegal. Error bars indicate +/− one standard error of mean (n=5).

**Table S6.** Nonlinear regression model for relative dry weight and nutrient mineralization (Ae ^̶ kt^) of leaves after 52 weeks in litterbags placed on the soil during the rainy season in Dahra in the Sahelian zone of Senegal. A stands for the labile fraction, k for the decay or nutrient release rate and t_50_ is the half-life in weeks. R² denotes the goodness of fit of the decay curve.

| Nutrient/Species | A | K | t_50_ | R² | F-value | *P*-value |
| --- | --- | --- | --- | --- | --- | --- |
| Dry weight |  |  |  |  |  |  |
| *F. albida* | 79.95 | 0.02 a | 34.66 | 0.60 | 4.990 | 0.016 |
| *G. senegalensis* | 83.82 | 0.01 b | 69.31 | 0.51 |  |  |
| *P. lucens* | 84.48 | 0.02 a | 34.66 | 0.70 |  |  |
| *P. reticulatum* | 81.37 | 0.01 b | 69.31 | 0.35 |  |  |
| Nitrogen |  |  |  |  |  |  |
| *F. albida* | 82.41 | 0.03 a | 23.10 | 0.61 | 5.860 | 0.0067 |
| *G. senegalensis* | 92.03 | 0.01 b | 69.31 | 0.44 |  |  |
| *P. lucens* | 85.25 | 0.02 a | 34.66 | 0.69 |  |  |
| *P. reticulatum* | 84.97 | 0.01 b | 69.31 | 0.36 |  |  |
| Phosphorus |  |  |  |  |  |  |
| *F. albida* | 78.82 | 0.09 a | 7.70 | 0.56 | 3.370 | 0.0446 |
| *G. senegalensis* | 79.89 | 0.03 b | 23.10 | 0.54 |  |  |
| *P. lucens* | 80.76 | 0.06 ab | 11.55 | 0.69 |  |  |
| *P. reticulatum* | 78.89 | 0.03 b | 23.10 | 0.52 |  |  |
| Potassium |  |  |  |  |  |  |
| *F. albida* | 90.85 | 0.13 a | 5.33 | 0.75 | 3.918 | 0.028 |
| *G. senegalensis* | 96.85 | 0.11 a | 6.30 | 0.88 |  |  |
| *P. lucens* | 92.63 | 0.12 a | 5.78 | 0.80 |  |  |
| *P. reticulatum* | 95.13 | 0.05 b | 13.86 | 0.63 |  |  |

Lowercase letters indicate significant differences between plant species (n = 4) means at *P* < 0.05.

**
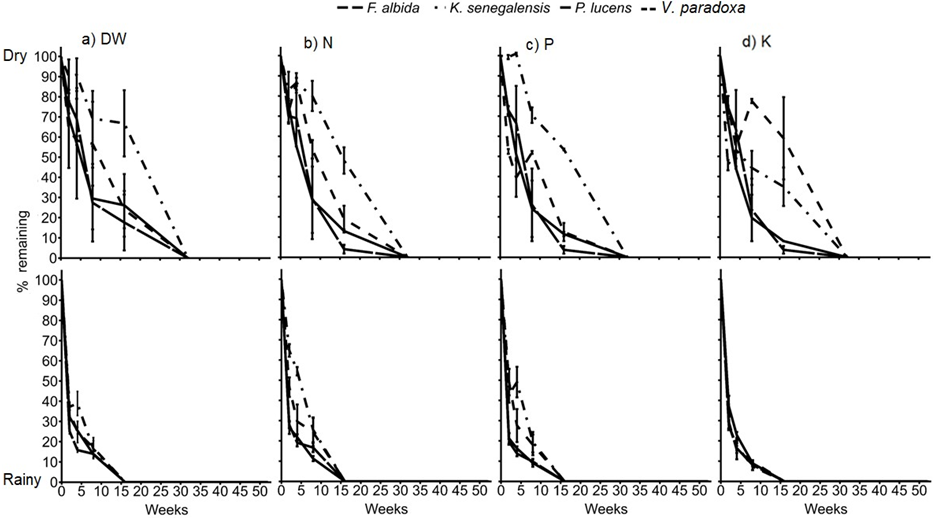
**

**Figure S2.** Decay and nutrient release patterns of different tree/shrub leaves after 52 weeks in litterbags placed on the soil across season in Katibougou in the Sudano-Sahelian zone of Koulikoro, Mali (rainy, n=5 and dry, n=4).

**Table S7.** Nonlinear regression model for relative dry weight and nutrient losses (Ae ^̶ kt^) of leaves after 52 weeks in litterbags placed on the soil across the dry and rainy seasons in Katibougou in the Sudano-Sahelian zone of Koulikoro, Mali. A stands for the labile fraction, k for the decay or nutrient release rate and t_50_ is the half-life in weeks. R² stands for the goodness of fit of the decay curve.

| Season | Nutrient/Species | A | K | t_50_ | R² | Two-way ANOVA | | |
| --- | --- | --- | --- | --- | --- | --- | --- | --- |
|  |  |  |  |  |  | Season | Species | Season × Species |
|  | Dry weight |  |  |  |  |  |  |  |
| Dry | *F. albida* | 99.60 | 0.12 ab | 5.99 | 0.83 | F = 45.893;  *P* < 0.001 | F = 3.609;  *P* = 0.027 | F = 2.046;  *P* = 0.132 |
|  | *K. senegalensis* | 105.65 | 0.06 a | 13.22 | 0.70 |  |  |  |
|  | *P. lucens* | 103.93 | 0.25 abc | 3.83 | 0.72 |  |  |  |
|  | *V. paradoxa* | 96.23 | 0.12 ab | 12.44 | 0.64 |  |  |  |
| Rainy | *F. albida* | 99.29 | 0.55 d | 1.31 | 0.96 |  |  |  |
|  | *K. senegalensis* | 94.85 | 0.30 abc | 2.44 | 0.92 |  |  |  |
|  | *P. lucens* | 86.38 | 0.40 bcd | 1.96 | 0.96 |  |  |  |
|  | *V. paradoxa* | 97.15 | 0.43 cd | 1.80 | 0.94 |  |  |  |
|  | Nitrogen |  |  |  |  |  |  |  |
| Dry | *F. albida* | 97.10 | 0.12 ab | 5.99 | 0.82 | F = 51.042;  *P* < 0.001 | F = 9.8575;  *P* < 0.001 | F = 3.252;  *P* = 0.038 |
|  | *K. senegalensis* | 102.61 | 0.06 a | 12.42 | 0.72 |  |  |  |
|  | *P. lucens* | 97.88 | 0.17 ab | 6.81 | 0.66 |  |  |  |
|  | *V. paradoxa* | 92.43 | 0.13 ab | 12.32 | 0.50 |  |  |  |
| Rainy | *F. albida* | 98.77 | 0.48 cd | 1.49 | 0.95 |  |  |  |
|  | *K. senegalensis* | 96.68 | 0.19 ab | 3.95 | 0.92 |  |  |  |
|  | *P. lucens* | 98.42 | 0.55 d | 1.31 | 0.95 |  |  |  |
|  | *V. paradoxa* | 96.09 | 0.30 bc | 2.74 | 0.93 |  |  |  |
|  | Phosphorus |  |  |  |  |  |  |  |
| Dry | *F. albida* | 98.86 | 0.13 ab | 5.61 | 0.85 | F = 49.6602;  *P* < 0.001 | F = 13.545;  *P* < 0.001 | F = 3.388;  *P* = 0.032 |
|  | *K. senegalensis* | 145.57 | 0.05 a | 14.15 | 0.47 |  |  |  |
|  | *P. lucens* | 97.31 | 0.30 ab | 3.40 | 0.72 |  |  |  |
|  | *V. paradoxa* | 85.05 | 0.19 ab | 8.64 | 0.59 |  |  |  |
| Rainy | *F. albida* | 99.56 | 0.66 c | 1.09 | 0.97 |  |  |  |
|  | *K. senegalensis* | 94.60 | 0.23 ab | 3.21 | 0.91 |  |  |  |
|  | *P. lucens* | 99.28 | 0.71 c | 1.01 | 0.96 |  |  |  |
|  | *V. paradoxa* | 99.37 | 0.37 b | 2.24 | 0.96 |  |  |  |
|  | Potassium |  |  |  |  |  |  |  |
| Dry | *F. albida* | 99.96 | 0.14 ab | 5.10 | 0.87 | F = 58.6733;  *P* < 0.001 | F = 0.2633;  *P* = 0.8528 | F = 1.675;  *P* = 0.197 |
|  | *K. senegalensis* | 91.08 | 0.12 a | 6.70 | 0.76 |  |  |  |
|  | *P. lucens* | 105.55 | 0.25 abc | 3.39 | 0.66 |  |  |  |
|  | *V. paradoxa* | 94.68 | 0.13 a | 10.45 | 0.73 |  |  |  |
| Rainy | *F. albida* | 100.50 | 0.49 cd | 1.56 | 0.95 |  |  |  |
|  | *K. senegalensis* | 98.53 | 0.55 d | 1.44 | 0.93 |  |  |  |
|  | *P. lucens* | 98.42 | 0.45 bcd | 1.62 | 0.96 |  |  |  |
|  | *V. paradoxa* | 99.32 | 0.59 d | 1.26 | 0.98 |  |  |  |

Lowercase letters indicate significant differences between plant species (n = 4) across season (n = 2) at *P* < 0.05.

**
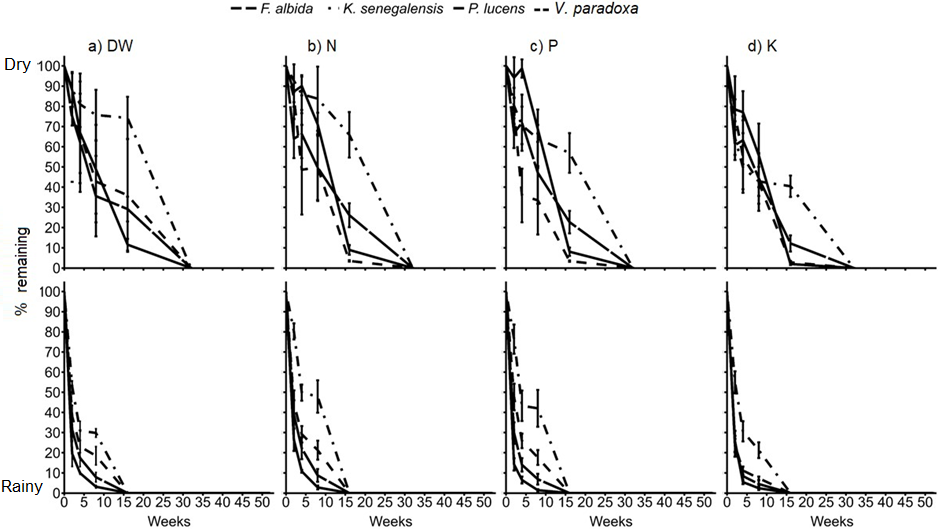
**

**Figure S3.** Decay and nutrient release patterns of different tree/shrub leaves after 52 weeks in litterbags placed on the soil across seasons in Katibougou in the Sudano-Sahelian zone of West Central, Burkina Faso (rainy, n=5 and dry, n=4).

**Table S8.** Nonlinear regression model for relative dry weight and nutrient losses (Ae ^̶ kt^) of leaves after 52 weeks in litterbags placed on the soil across the dry and rainy seasons in Saria in the Sudano-Sahelian zone of West Central, Burkina Faso. A stands for the labile fraction, k for the decay or nutrient release rate and t t_50_ is the half-life in weeks. R² denotes the goodness of fit of the decay curve.

| Season | Nutrient/Species | A | K | t_50_ | R² | Two-way ANOVA | | |
| --- | --- | --- | --- | --- | --- | --- | --- | --- |
|  |  |  |  |  |  | Season | Species | Season × Species |
|  | Dry weight |  |  |  |  |  |  |  |
| Dry | *F. albida* | 97.52 | 0.11 a | 6.30 | 0.73 | F = 90.062;  *P* < 001 | F = 11.680;  *P* < 001 | F = 4.0049;  *P* = 0.018 |
|  | *K. senegalensis* | 101.60 | 0.04 a | 17.33 | 0.57 |  |  |  |
|  | *P. lucens* | 108.91 | 0.15 a | 4.62 | 0.85 |  |  |  |
|  | *V. paradoxa* | 107.06 | 0.10 a | 6.93 | 0.71 |  |  |  |
| Rainy | *F. albida* | 99.37 | 0.53 ab | 1.31 | 0.96 |  |  |  |
|  | *K. senegalensis* | 96.17 | 0.25 a | 2.77 | 0.95 |  |  |  |
|  | *P. lucens* | 99.90 | 0.72 b | 0.96 | 0.99 |  |  |  |
|  | *V. paradoxa* | 97.95 | 0.39 ab | 1.78 | 0.93 |  |  |  |
|  | Nitrogen |  |  |  |  |  |  |  |
| Dry | *F. albida* | 94.46 | 0.10 a | 6.93 | 0.67 | F = 15.398;  *P* < 001 | F = 6.766;  *P* = 0.002 | F = 3.437;  *P* = 0.032 |
|  | *K. senegalensis* | 108.62 | 0.04 a | 17.33 | 0.58 |  |  |  |
|  | *P. lucens* | 108.21 | 0.16 a | 4.33 | 0.83 |  |  |  |
|  | *V. paradoxa* | 107.72 | 0.28 ab | 2.48 | 0.69 |  |  |  |
| Rainy | *F. albida* | 99.22 | 0.40 ab | 1.73 | 0.98 |  |  |  |
|  | *K. senegalensis* | 99.70 | 0.13 a | 5.33 | 0.91 |  |  |  |
|  | *P. lucens* | 99.91 | 0.68 b | 1.02 | 0.99 |  |  |  |
|  | *V. paradoxa* | 98.49 | 0.33 ab | 2.10 | 0.95 |  |  |  |
|  | Phosphorus |  |  |  |  |  |  |  |
| Dry | *F. albida* | 95.37 | 0.11 ab | 6.30 | 0.68 | F = 19.98;  *P* < 001 | F = 9.250;  *P* < 001 | F = 7.156;  *P* = 0.001 |
|  | *K. senegalensis* | 96.12 | 0.05 a | 13.86 | 0.65 |  |  |  |
|  | *P. lucens* | 111.45 | 0.15 ab | 4.62 | 0.82 |  |  |  |
|  | *V. paradoxa* | 96.75 | 0.34 ab | 2.04 | 0.65 |  |  |  |
| Rainy | *F. albida* | 99.48 | 0.53 b | 1.31 | 0.98 |  |  |  |
|  | *K. senegalensis* | 97.64 | 0.15 ab | 4.62 | 0.88 |  |  |  |
|  | *P. lucens* | 99.97 | 1.00 c | 0.69 | 0.99 |  |  |  |
|  | *V. paradoxa* | 98.49 | 0.33 ab | 2.10 | 0.95 |  |  |  |
|  | Potassium |  |  |  |  |  |  |  |
| Dry | *F. albida* | 92.52 | 0.15 ab | 4.62 | 0.77 | F = 18.437;  *P* < 001 | F = 3.8007;  *P* = 0.023 | F = 1.109;  *P* = 0.364 |
|  | *K. senegalensis* | 87.70 | 0.11 a | 6.30 | 0.56 |  |  |  |
|  | *P. lucens* | 105.18 | 0.19 ab | 3.65 | 0.83 |  |  |  |
|  | *V. paradoxa* | 96.33 | 0.45 ab | 1.54 | 0.68 |  |  |  |
| Rainy | *F. albida* | 99.85 | 0.71 b | 0.98 | 0.99 |  |  |  |
|  | *K. senegalensis* | 99.04 | 0.30 ab | 2.31 | 0.94 |  |  |  |
|  | *P. lucens* | 100.12 | 0.73 b | 0.95 | 0.99 |  |  |  |
|  | *V. paradoxa* | 99.88 | 0.73 b | 0.95 | 0.98 |  |  |  |

Lowercase letters indicate significant differences between plant species (N = 4) across seasons (n = 2) at *P* < 0.05.
